# The spectral norms of geometric circulant matrices with the generalized *k*-Horadam numbers

**DOI:** 10.1186/s13660-017-1608-4

**Published:** 2018-01-10

**Authors:** Baijuan Shi

**Affiliations:** School of Mathematics, Northwest University, Xi’an, Shaanxi P.R. China

**Keywords:** geometric circulant matrix, *r*-circulant matrix, generalized *k*-Horadam numbers, spectral norms, Euclidean norms

## Abstract

In this paper, we use the algebra methods, the properties of the *r*-circulant matrix and the geometric circulant matrix to study the upper and lower bound estimate problems for the spectral norms of a geometric circulant matrix involving the generalized *k*-Horadam numbers, and we obtain some sharp estimations for them. We can also give a new estimation for the norms of a *r*-circulant matrix involving the generalized *k*-Horadam numbers.

## Introduction

Let $n\geq2$ be an integer, *r* be any real or complex number. Then a $n\times n$ *r*-circulant matrix $C_{r}$ is defined by

$$ C_{r}=\left ( \textstyle\begin{array}{c@{\quad}c@{\quad}c@{\quad}c@{\quad}c@{\quad}c} c_{0}&c_{1}&c_{1}&\cdots&c_{n-2}&c_{n-1}\\ rc_{n-1}&c_{0}&c_{1}&\cdots&c_{n-3}&c_{n-2}\\ rc_{n-2}&rc_{n-1}&c_{0}&\cdots&c_{n-4}&c_{n-3}\\ \vdots&\vdots&\vdots& &\vdots&\vdots\\ rc_{1}&rc_{2}&rc_{3}&\cdots&rc_{n-1}&c_{0} \end{array}\displaystyle \right )_{n\times n}. $$

This matrix was first proposed by Davis in [1], then one found it has many interesting properties, and it is one of the most important research subject in the field of the computation and pure mathematics (see [2–9]). For example, Shen and Cen [3] studied the norms of *r*-circulant matrices with Fibonacci and Lucas numbers. Afterward, Kızılateş and Tuglu [10] defined a new geometric circulant matrix,

$$ C_{r^{*}}=\left ( \textstyle\begin{array}{c@{\quad}c@{\quad}c@{\quad}c@{\quad}c@{\quad}c} c_{0}&c_{1}&c_{1}&\cdots&c_{n-2}&c_{n-1}\\ rc_{n-1}&c_{0}&c_{1}&\cdots&c_{n-3}&c_{n-2}\\ r^{2}c_{n-2}&rc_{n-1}&c_{0}&\cdots&c_{n-4}&c_{n-3}\\ \vdots&\vdots&\vdots& &\vdots&\vdots\\ r^{n-1}c_{1}&r^{n-2}c_{2}&r^{n-3}c_{3}&\cdots&rc_{n-1}&c_{0} \end{array}\displaystyle \right )_{n\times n}, $$

then they found the bounds for the spectral norms of geometric circulant matrices with the generalized Fibonacci number and Lucas numbers. Obviously, $C_{r}$ and $C_{r^{*}}$ are determined by the parameter *r* and the first row elements of the matrix. When the parameter satisfies $r=1$, we get the classical circulant matrix.

The *r*-circulant matrix and geometric circulant matrix with some commonly chosen values have also been studied by many researchers in recent years. Particularly, many scholars have learned the norms of those matrices based on the special properties. For example, many scholars have studied the spectral norms of circulant matrices and *r*-circulant matrices with famous sequences [2–6]. In [7], Kocer *et al.* have obtained the norms of semicirculant and circulant matrices with Horadam numbers. In [8], Yazlik and Taskara have studied eigenvalues, determinant and the spectral norms of circulant matrix involving the generalized *k*-Horadam numbers. In [9], Raza *et al.* have also studied the norms of many special matrices with generalized Fibonacci sequences and generalized Pell-Padovan. In [10], Can and Naim have defined geometric circulant matrices and studied the bounds for the spectral norms of geometric circulant matrices involving the generalized Fibonacci number and Lucas numbers. Some great contributions for the spectral norms of *r*-circulant matrix and geometric circulant matrix can be found in references [11–17]. In particular, Köme and Yazlik [11] have presented new upper and lower bounds for the spectral norms of the *r*-circulant matrices with biperiodic Fibonacci and biperiodic Lucas numbers, $Q=C_{r}( (\frac{b}{a})^{\frac{\xi (1)}{2}}q_{0}, (\frac{b}{a})^{\frac{\xi(2)}{2}}q_{1}, (\frac {b}{a})^{\frac{\xi(3)}{2}}q_{2},\ldots, (\frac{b}{a})^{\frac{\xi (n)}{2}}q_{n-1})$, and $L=C_{r}( (\frac{b}{a})^{\frac{\xi(0)}{2}}l_{0}, (\frac {b}{a})^{\frac{\xi(1)}{2}}l_{1}, (\frac{b}{a})^{\frac{\xi (2)}{2}}l_{2},\ldots, (\frac{b}{a})^{\frac{\xi(n-1)}{2}}l_{n-1})$, then they gave the lower and upper bounds for the spectral norms of Kronecker and Hadamard products of the matrices *Q* and *L*.

Considering the above articles, on the one hand, we obtain a new lower and upper bounds estimates for the spectral norms of the geometric circulant matrix with the generalized *k*-Horadam numbers, at the same time, we also get the spectral norms of geometric circulant matrices involving all second-order recurrence sequences or polynomials. For example, we get the bounds for the spectral norms of geometric circulant matrices involving the generalized Fibonacci number and Lucas numbers [10]. On the other hand, we improve the results in reference [18], and we give new and better upper bounds for the norms of an *r*-circulant matrix involving the generalized *k*-Horadam numbers.

## Preliminaries

### Definition 1

The generalized *k*-Horadam numbers have the form (see [18])

1 $$ H_{k,n+2}=f(k)H_{k,n+1}+g(k)H_{k,n},\qquad H_{k,0}=a,\qquad H_{k,0}=b, $$

where positive integer $n\geq0$, $f^{2}(k)+4g(k)>0$. Clearly, the form is a general shape of the special second-order sequences or polynomials. For example, let $f(k)=g(k)=1$, $a=0$, and $b=1$, then we get the famous Fibonacci number. Let $f(k)=g(k)=1$, $a=2$, and $b=1$, the Lucas number is obtained. Let $f(x)=2x$, $g(x)=-1$, $a=1$, and $b=x$, then we have the Chebyshev polynomial. Namely, if we take an appropriate value with $f(k)$, $g(k)$, *a*, *b* in (1), then we get the familiar all second-order sequences.

For the sequence $H_{k,n}$, the characteristic equation $x^{2}-f(k)x-g(k)=0$ has two distinct roots, denoted by *α*, *β*. The Binet formula $H_{k,n}$ can be represented by $H_{k,n}=\frac{X\alpha ^{n}-Y\beta^{n}}{\alpha-\beta}$, where $X=b-a\beta$, $Y=b-a\alpha$.

### Definition 2

The geometric circulant matrix $C_{r^{*}}$ is defined by (see [10])

$$ C_{r^{*}}=\left ( \textstyle\begin{array}{c@{\quad}c@{\quad}c@{\quad}c@{\quad}c@{\quad}c} c_{0}&c_{1}&c_{1}&\cdots&c_{n-2}&c_{n-1}\\ rc_{n-1}&c_{0}&c_{1}&\cdots&c_{n-3}&c_{n-2}\\ r^{2}c_{n-2}&rc_{n-1}&c_{0}&\cdots&c_{n-4}&c_{n-3}\\ \vdots&\vdots&\vdots& &\vdots&\vdots\\ r^{n-1}c_{1}&r^{n-2}c_{2}&r^{n-3}c_{3}&\cdots&rc_{n-1}&c_{0} \end{array}\displaystyle \right )_{n\times n}. $$

We denote it for convenience by $C_{r^{*}}=\operatorname{Circ}_{r^{*}}(c_{0},c_{1},c_{2},\ldots ,c_{n-1})$. When the parameter $r=1$, the geometric circulant matrix turns into a circulant matrix.

### Definition 3

Let us take any matrix $A=(a_{ij})\in M_{m\times n}(C)$, the spectral norm and the Euclidean norm of the matrix *A* are

$$\begin{gathered} \|A\|_{2}=\sqrt{ \max_{1\leq i\leq n}\lambda_{i} \bigl(A^{H}A\bigr)} ,\qquad \|A\|_{E}= \Biggl( \sum^{m}_{i=1} \sum^{n}_{j=1}|a_{ij}|^{2} \Biggr)^{\frac{1}{2}},\end{gathered} $$

respectively, where $\lambda_{i}(A^{H}A)$ is the eigenvalue of $A^{H}A$ and $A^{H}$ is the conjugate transpose of matrix *A*.

The following inequalities hold between the Euclidean norm and spectral norm (see [19]):

2 $$ \frac{1}{\sqrt{n}}\|A\|_{E}\leq\|A\|_{2}\leq\|A \|_{E},\qquad \|A\|_{2}\leq\|A\| _{E}\leq\sqrt{n}\|A \|_{2}. $$

### Definition 4

Let $A=(a_{ij})$ and $B=(b_{ij})$ be $m\times n$ matrices, then the Hadamard product of *A* and *B* is the $m\times n$ matrix of elementwise products, namely $A\circ B=(a_{ij}b_{ij})$.

Then we have the following inequalities (see [20]):

3 $$ \|A\circ B\|_{2}\leq r_{1}(A)C_{1}(B), $$

where $r_{1}(A)= \max_{1\leq i\leq m}\sqrt{\sum^{n}_{j=1}|a_{ij}|^{2}}$, $C_{1}(B)= \max_{1\leq j\leq n}\sqrt{ \sum^{m}_{i=1}|b_{ij}|^{2}}$.

### Lemma 1

*Let* $\Theta_{r}$ *be a basic* *r*-*circulant matrix*, $\Theta_{r}=C_{r}(0,1,0,\ldots,0)$, *considering the structure of the power of* $\Theta_{r}$, *clearly*, $\Theta_{r}^{n}=rI_{n}$, *and* $C_{r}=f(\Theta_{r})= \sum^{n-1}_{k=0}c_{k}\Theta_{r}^{i}$, *where* $f(x)= \sum^{n-1}_{k=0}c_{k}x^{i}$.

### Lemma 2

*For any positive integer* $n\geq1$, *we can easily get*

4 $$ \sum^{n-1}_{i=0}H_{k,i}= \frac{a+b-af(k)-H_{k,n}-g(k)H_{k,n-1}}{1-f(k)-g(k)}. $$

### Lemma 3

*For any positive integer* $n\geq1$,

5 $$ \sum^{n-1}_{i=0} \biggl(\frac{H_{k,i}}{|r|^{i}} \biggr)^{2}=\frac{N+\frac {H^{2}_{k,n}}{|r|^{2n-4}}-\frac {g^{2}(k)}{|r|^{2n}}H^{2}_{k,n-1}}{f^{2}(k)+2g(k)-\frac{g^{2}(k)}{|r|^{2}}-|r|^{2}}, $$

*where* $N= (b-af(k) )^{2}-a^{2}|r|^{2}-2XY (\frac{1- (-\frac {g(k)}{|r|^{2}} )^{n}}{1+\frac{g(k)}{|r|^{2}}} )$.

### Proof

Let $A= \sum^{n-1}_{i=0} (\frac {H_{k,i}}{|r|^{i}} )^{2}$, by the definition of $H_{k,i}$, we get

$$\begin{gathered} A= \sum^{n-1}_{i=0}\frac {H^{2}_{k,i+1}+g^{2}(k)H^{2}_{k,i-1}-2g(k)H_{k,i+1}H_{k,i-1}}{|r|^{2i}f^{2}(k)}, \\ \begin{aligned} \sum^{n-1}_{i=0} \frac{H_{k,i+1}H_{k,i-1}}{|r|^{2i}}&= \sum^{n-1}_{i=0} \frac{X^{2}\alpha^{2i}+Y^{2}\beta^{2i}-XY\alpha^{i+1}\beta ^{i-1}-XY\alpha^{i-1}\beta^{i+1}}{ (\alpha-\beta )^{2}|r|^{2i}} \\ &= \sum^{n-1}_{i=0}\frac{ (X\alpha^{i}-Y\beta^{i} )^{2}}{ (\alpha -\beta )^{2}|r|^{2i}}-XY \sum ^{n-1}_{i=0}\frac{ (\alpha\beta )^{i-1}}{|r|^{2i}} \\ &=A+\frac{XY}{g(k)}\ast\frac{1- (-\frac{g(k)}{|r|^{2}} )^{n}}{1+\frac {g(k)}{|r|^{2}}}, \end{aligned} \\ \frac{1}{|r|^{2}} \sum^{n-1}_{i=0} \biggl( \frac{H_{k,i+1}}{|r|^{i}} \biggr)^{2}=A-H^{2}_{k,0}+ \biggl( \frac{H_{k,n}}{|r|^{n}} \biggr)^{2}, \\ |r|^{2} \sum^{n-1}_{i=0} \biggl( \frac{H_{k,i-1}}{|r|^{i}} \biggr)^{2}=A+ \biggl(\frac{H_{k,-1}}{|r|^{-1}} \biggr)^{2}- \biggl(\frac {H_{k,n-1}}{|r|^{n-1}} \biggr)^{2}, \\ \begin{aligned}Af^{2}(k)={}&A \biggl(|r|^{2}+\frac{g^{2}(k)}{|r|^{2}}-2g(k) \biggr)+ \bigl(b-af(k) \bigr)^{2}-a^{2}|r|^{2}+ \frac{H^{2}_{k,n}}{|r|^{2n-4}} \\ &-\frac{g^{2}(k)}{|r|^{2n}}H^{2}_{k,n-1} -2XY \biggl(\frac{1- (-\frac{g(k)}{|r|^{2}} )^{n}}{1+\frac {g(k)}{|r|^{2}}} \biggr).\end{aligned}\end{gathered} $$

So,

$$\sum^{n-1}_{i=0} \biggl(\frac{H_{k,i}}{|r|^{i}} \biggr)^{2}=\frac{N+\frac {H^{2}_{k,n}}{|r|^{2n-4}}-\frac {g^{2}(k)}{|r|^{2n}}H^{2}_{k,n-1}}{f^{2}(k)+2g(k)-\frac{g^{2}(k)}{|r|^{2}}-|r|^{2}}, $$

where $N= (b-af(k) )^{2}-a^{2}|r|^{2}-2XY (\frac{1- (-\frac {g(k)}{|r|^{2}} )^{n}}{1+\frac{g(k)}{|r|^{2}}} )$. Let $r=1$, we get

$$ \sum^{n-1}_{i=0}H^{2}_{k,i}= \frac{H^{2}_{k,n}-g^{2}(k)H^{2}_{k,n-1}+ (b-af(k) )^{2}-a^{2}-2XY (\frac{1- (-g(k) )^{n}}{1+g(k)} )}{f^{2}(k)-g^{2}(k)+2g(k)-1}, $$

where $X=b-a\beta$, $Y=b-a\alpha$. □

## Main results

### Theorem 1

*Let* $H_{r^{*}}=\operatorname{Circ}_{r^{*}}(H_{k,0},H_{k,1},H_{k,2},\ldots,H_{k,n-1})$ *be an* $n\times n$ *geometric circulant matrix*, *completely defined by generalized* *k*-*Horadam numbers*.

(i) *If* $|r|>1$, *then* $\sqrt{ \sum^{n-1}_{i=0}H_{k,i}^{2}}\leq\| H_{r^{*}}\|_{2}\leq\sqrt{\frac{1-|r|^{2n}}{1-|r|^{2}} \sum^{n-1}_{i=0}H_{k,i}^{2}}$.
(ii) *If* $|r|<1$, *then* $\sqrt{\frac {N|r|^{2n}+|r|^{4}H^{2}_{k,n}-g^{2}(k)H^{2}_{k,n-1}}{f^{2}(k)+2g(k)-\frac {g^{2}(k)}{|r|^{2}}-|r|^{2}}}\leq\|H_{r^{*}}\|_{2}\leq\sqrt{n \sum^{n-1}_{i=0}H^{2}_{k,i}}$, *where* $N= (b-af(k) )^{2}-a^{2}|r|^{2}-2XY (\frac{1- (-\frac {g(k)}{|r|^{2}} )^{n}}{1+\frac{g(k)}{|r|^{2}}} )$.

### Proof

$$ H_{r^{*}}=\left ( \textstyle\begin{array}{c@{\quad}c@{\quad}c@{\quad}c@{\quad}c@{\quad}c} H_{k,0}&H_{k,1}&H_{k,2}&\cdots&H_{k,n-2}&H_{k,n-1}\\ rH_{k,n-1}&H_{k,0}&H_{k,1}&\cdots&H_{k,n-3}&H_{k,n-2}\\ r^{2}H_{k,n-2}&rH_{k,n-1}&H_{k,0}&\cdots&H_{k,n-4}&H_{k,n-3}\\ \vdots&\vdots&\vdots& &\vdots&\vdots\\ r^{n-1}H_{k,1}&r^{n-2}H_{k,2}&r^{n-3}H_{k,3}&\cdots&rH_{k,n-1}&H_{k,0} \end{array}\displaystyle \right )_{n\times n}. $$

(i) From $|r|>1$ and by using the definition of Euclidean norm, we have

$$ \begin{aligned} \|H_{r^{*}}\|^{2}_{E}&= \sum^{n-1}_{i=0} (n-i )H^{2}_{k,i}+ \sum^{n-1}_{i=1}i\big|r^{n-i}\big|^{2}H^{2}_{k,i} \\ &\geq\sum_{i=0}^{n-1} (n-i )H^{2}_{k,i}+\sum^{n-1}_{i=1}iH^{2}_{k,i} \\ &=n\sum^{n-1}_{i=0}H^{2}_{k,i}. \end{aligned} $$

That is,

$$\frac{1}{\sqrt{n}}\|H_{r^{*}}\|_{E}\geq\sqrt{\sum ^{n-1}_{i=0}H^{2}_{k,i}}, $$

from (2), we have $\sqrt{ \sum^{n-1}_{k=0}H^{2}_{k,i}}\leq\|H_{r^{*}}\|_{2}$.

Let the matrices *A* and *B* be presented by

$$ A=\left ( \textstyle\begin{array}{c@{\quad}c@{\quad}c@{\quad}c@{\quad}c@{\quad}c} 1&1&1&\cdots&1&1\\ r&1&1&\cdots&1&1\\ r^{2}&r&1&\cdots&1&1\\ \vdots&\vdots&\vdots& &\vdots&\vdots\\ r^{n-1}&r^{n-2}&r^{n-3}&\cdots&r&1 \end{array}\displaystyle \right )_{n\times n} $$

and

$$ B=\left ( \textstyle\begin{array}{c@{\quad}c@{\quad}c@{\quad}c@{\quad}c@{\quad}c} H_{k,0}&H_{k,1}&H_{k,2}&\cdots&H_{k,n-2}&H_{k,n-1}\\ H_{k,n-1}&H_{k,0}&H_{k,1}&\cdots&H_{k,n-3}&H_{k,n-2}\\ H_{k,n-2}&H_{k,n-1}&H_{k,0}&\cdots&H_{k,n-4}&H_{k,n-3}\\ \vdots&\vdots&\vdots& &\vdots&\vdots\\ H_{k,1}&H_{k,2}&H_{k,3}&\cdots&H_{k,n-1}&H_{k,0} \end{array}\displaystyle \right )_{n\times n}, $$

then $H_{r^{*}}=A\circ B$. So $\|H_{r^{*}}\|_{2}=\|A\circ B\|_{2}\leq r_{1}(A)C_{1}(B)$,

$$\begin{gathered} \begin{aligned} r_{1}(A)&=\max_{1\leq i\leq n} \sqrt{\sum_{j=1}^{n}|a_{ij}|^{2}}= \sqrt{\sum_{j=1}^{n}|a_{nj}|^{2}} \\ &=\sqrt{1+|r|^{2}+\cdots+\big|r^{n-1}\big|^{2}}=\sqrt{ \frac{1-|r|^{2n}}{1-|r|^{2}}}, \end{aligned} \\c_{1}(B)=\max_{1\leq j\leq n}\sqrt{\sum _{i=1}^{n}|b_{ij}|^{2}}=\sqrt {\sum_{j=1}^{n}|b_{in}|^{2}}= \sqrt{ \sum^{n-1}_{i=0}H^{2}_{k,i}}.\end{gathered} $$

Therefore, we have

$$\|H_{r^{*}}\|_{2}\leq r_{1}(A)c_{1}(B)= \sqrt{\frac{1-|r|^{2n}}{1-|r|^{2}} \sum^{n-1}_{i=0}H^{2}_{k,i}}. $$

Thus, we can obtain the inequality

$$\sqrt{ \sum^{n-1}_{i=0}H^{2}_{k,i}} \leq\|H_{r^{*}}\|_{2}\leq\sqrt{\frac {1-|r|^{2n}}{1-|r|^{2}} \sum ^{n-1}_{i=0}H^{2}_{k,i}}. $$

(ii) From $|r|<1$,

$$ \begin{aligned} \|H_{r^{*}}\|^{2}_{E}&= \sum^{n-1}_{i=0} (n-i )H^{2}_{k,i}+ \sum^{n-1}_{i=1}i\big|r^{n-i}\big|^{2}H^{2}_{k,i} \\ &\geq\sum_{i=0}^{n-1} (n-i )\big|r^{n-i}\big|^{2}H^{2}_{k,i}+\sum ^{n-1}_{i=1}i\big|r^{n-i}\big|^{2}H^{2}_{k,i} \\ &=n|r|^{2n}\sum^{n-1}_{i=0} \biggl( \frac{H_{k,i}}{|r|^{i}} \biggr)^{2}. \end{aligned} $$

By using (5), we get

$$\sqrt{\frac {N|r|^{2n}+|r|^{4}H^{2}_{k,n}-g^{2}(k)H^{2}_{k,n-1}}{f^{2}(k)+2g(k)-\frac {g^{2}(k)}{|r|^{2}}-|r|^{2}}}\leq\|H_{r^{*}}\|_{2}. $$

For the matrices *A* and *B* as mentioned above we have

$$ A=\left ( \textstyle\begin{array}{c@{\quad}c@{\quad}c@{\quad}c@{\quad}c@{\quad}c} 1&1&1&\cdots&1&1\\ r&1&1&\cdots&1&1\\ r^{2}&r&1&\cdots&1&1\\ \vdots&\vdots&\vdots& &\vdots&\vdots\\ r^{n-1}&r^{n-2}&r^{n-3}&\cdots&r&1 \end{array}\displaystyle \right )_{n\times n} $$

and

$$ B=\left ( \textstyle\begin{array}{c@{\quad}c@{\quad}c@{\quad}c@{\quad}c@{\quad}c} H_{k,0}&H_{k,1}&H_{k,2}&\cdots&H_{k,n-2}&H_{k,n-1}\\ H_{k,n-1}&H_{k,0}&H_{k,1}&\cdots&H_{k,n-3}&H_{k,n-2}\\ H_{k,n-2}&H_{k,n-1}&H_{k,0}&\cdots&H_{k,n-4}&H_{k,n-3}\\ \vdots&\vdots&\vdots& &\vdots&\vdots\\ H_{k,1}&H_{k,2}&H_{k,3}&\cdots&H_{k,n-1}&H_{k,0} \end{array}\displaystyle \right )_{n\times n}. $$

In this case, $H_{r^{*}}=A\circ B$. So $\|H_{r^{*}}\|_{2}=\|A\circ B\|_{2}\leq r_{1}(A)C_{1}(B)$,

$$ \begin{gathered} r_{1}(A)=\max_{1\leq i\leq n} \sqrt{\sum_{j=1}^{n}|a_{ij}|^{2}}= \sqrt{n}, \\ c_{1}(B)=\max_{1\leq j\leq n}\sqrt{\sum _{i=1}^{n}|b_{ij}|^{2}}= \sqrt{\sum_{j=1}^{n}|b_{in}|^{2}}= \sqrt{ \sum^{n-1}_{i=0}H^{2}_{k,i}}, \\ \|H_{r^{*}}\|_{2}\leq\sqrt{n \sum ^{n-1}_{i=0}H^{2}_{k,i}}. \end{gathered} $$

Therefore, we have

$$\sqrt{\frac {N|r|^{2n}+|r|^{4}H^{2}_{k,n}-g^{2}(k)H^{2}_{k,n-1}}{f^{2}(k)+2g(k)-\frac {g^{2}(k)}{|r|^{2}}-|r|^{2}}}\leq\|H_{r^{*}}\|_{2}\leq \sqrt{n \sum^{n-1}_{i=0}H^{2}_{k,i}}, $$

where $N= (b-af(k) )^{2}-a^{2}|r|^{2}-2XY (\frac{1- (-\frac {g(k)}{|r|^{2}} )^{n}}{1+\frac{g(k)}{|r|^{2}}} )$. □

### Theorem 2

*Let* $H_{r}=\operatorname{Circ}_{r}(H_{k,0},H_{k,1},H_{k,2},\ldots ,H_{k,n-1})$ *be an* $n\times n$ *r*-*circulant matrix*.

(i) *If* $|r|>1$, *then*
  $$\sqrt{ \sum^{n-1}_{i=0}H^{2}_{k,i}} \leq\|H_{r}\|_{2}\leq\frac{a-|r| (af(k)-b )-|r|^{n}H_{k,n}-g(k)|r|^{n+1}H_{k,n-1}}{1-|r|f(k)-|r|^{2}g(k)}. $$
(ii) *If* $|r|<1$, *then*
  $$|r|\sqrt{ \sum^{n-1}_{i=0}H^{2}_{k,i}} \leq\|H_{r}\|_{2}\leq \sum^{n-1}_{i=0}H_{k,i}. $$

### Proof

$$ H_{r}=\left ( \textstyle\begin{array}{c@{\quad}c@{\quad}c@{\quad}c@{\quad}c@{\quad}c} H_{k,0}&H_{k,1}&H_{k,2}&\cdots&H_{k,n-2}&H_{k,n-1}\\ rH_{k,n-1}&H_{k,0}&H_{k,1}&\cdots&H_{k,n-3}&H_{k,n-2}\\ rH_{k,n-2}&rH_{k,n-1}&H_{k,0}&\cdots&H_{k,n-4}&H_{k,n-3}\\ \vdots&\vdots&\vdots& &\vdots&\vdots\\ rH_{k,1}&rH_{k,2}&rH_{k,3}&\cdots&rH_{k,n-1}&H_{k,0} \end{array}\displaystyle \right )_{n\times n}. $$

For $|r|>1$, from the definition of a Euclidean norm we have

$$ \|H_{r}\|^{2}_{E}= \sum ^{n-1}_{i=0} (n-i )H^{2}_{k,i}+\sum ^{n-1}_{i=1}i|r|^{2}H^{2}_{k,i} \geq n\sum^{n-1}_{i=0}H^{2}_{k,i}. $$

In this case,

$$\frac{1}{\sqrt{n}}\|H_{r}\|_{E}\geq\sqrt{\sum ^{n-1}_{i=0}H^{2}_{k,i}}, $$

then by using (2), we have $\sqrt{ \sum^{n-1}_{i=0}H^{2}_{k,i}}\leq\| H_{r}\|_{2}$.

For $|r|<1$,

$$ \|H_{r}\|^{2}_{E}= \sum ^{n-1}_{i=0} (n-i )H^{2}_{k,i}+\sum ^{n-1}_{i=1}i|r|^{2}H^{2}_{k,i} \geq n|r|^{2}\sum^{n-1}_{i=0}H^{2}_{k,i}. $$

So,

$$\frac{1}{\sqrt{n}}\|H_{r}\|_{E}\geq|r|\sqrt{\sum ^{n-1}_{i=0}H^{2}_{k,i}}. $$

By Lemma 1, we get $H_{r}=f(\Theta_{r})= \sum^{n-1}_{i=0}H_{k,i}\Theta _{r}^{i}$, where $f(x)= \sum^{n-1}_{i=0}H_{k,i}x^{i}$.

In this case,

$$\|H_{r}\|_{2}=\Bigg\| \sum^{n-1}_{i=0}H_{k,i} \Theta_{r}^{i}\Bigg\| _{2}\leq \sum ^{n-1}_{i=0}H_{k,i}\|\Theta_{r} \|^{i}_{2}, $$

because $\Theta_{r}^{H}\Theta_{r}$ has the form

$$ \Theta_{r}^{H}\Theta_{r}=\left ( \textstyle\begin{array}{c@{\quad}c@{\quad}c@{\quad}c@{\quad}c@{\quad}c} |r|^{2}&0&0&\cdots&0&0\\ 0&1&0&\cdots&0&0\\ 0&0&1&\cdots&0&0\\ \vdots&\vdots&\vdots& &\vdots&\vdots\\ 0&0&0&\cdots&0&1 \end{array}\displaystyle \right )_{n\times n}. $$

So for $|r|>1$, $\|\Theta_{r}\|_{2}=|r|$; for $|r|<1$, $\|\Theta_{r}\|_{2}=1$, and when $|r|>1$, we get

$$ \begin{aligned} \|H_{r}\|_{2}&\leq \sum ^{n-1}_{i=0}H_{k,i}\|\Theta_{r} \|^{i}_{2}= \sum^{n-1}_{i=0}|r|^{i}H_{k,i} \\ &=\frac{a-|r| (af(k)-b )-|r|^{n}H_{k,n}-g(k)|r|^{n+1}H_{k,n-1}}{1-|r|f(k)-|r|^{2}g(k)}, \end{aligned} $$

when $|r|<1$, $\|H_{r}\|_{2}\leq \sum^{n-1}_{i=0}H_{k,i}$. □

### Some notes

The methods of this paper can be deduced on the norms of *r*-circulant matrix, circulant matrix, geometric circulant matrices in particular with all second-order sequences, leading to better estimations than in reference [18].
